#  Technical recommendations for artificial insemination in sheep

**DOI:** 10.21451/1984-3143-AR2018-0129

**Published:** 2019-11-18

**Authors:** Alejandro Eduardo Gibbons, Jimena Fernandez, María Macarena Bruno-Galarraga, María Victoria Spinelli, Marcela Isabel Cueto

**Affiliations:** 1 Instituto Nacional de Tecnología Agropecuaria, Estación Experimental Agropecuaria Bariloche, Grupo de Reproducción en Rumiantes Menores, Bariloche, Argentina; 2 Universidad Nacional Lomas de Zamora, Facultad de Ciencias Agrarias, Buenos Aires, Argentina

**Keywords:** reproductive biotechnologies, ovis aries, spermatozoa, transcervical

## Abstract

Artificial insemination (AI) was the first important biotechnology applied to improve the genetics of farm animals. It allows the rapid and massive diffusion of desirable characteristics of males with high productive potential. We describe the different types of estrus induction and synchronization techniques and the use of the AI with fresh, chilled or frozen semen. Through the adequacy of the protocols of estrus synchronization and AI to the different production systems, the efficient use of reproductive techniques is possible, reaching acceptable pregnancy rates. Summary of reproductive results obtained using cervical and laparoscopic AI are presented.

## Introduction

The artificial insemination (AI) technique is an assisted reproduction technology and consists in the application of a dose of semen into the female reproductive tract by means of instruments. In combination with other technologies such as induction/synchronization of estrus and ovulation, AI can add genetic value to livestock by spreading the use of males of high productive characteristics (Alvares et al., 2015). Moreover, it makes possible the use of death, old or injured males and can prevent the transmission of sexual diseases (Hernández Ballesteros et al., 2015). The AI in the ovine species and cryopreservation of ram’s semen were comprehensively reviewed (Evans and Maxwell, 1987; Chemineau et al., 1991; Salamon and Maxwell, 2000; Parkinson, 2009).

In some countries, AI has had a great development and has served as a fundamental tool for genetic improvement in sheep. More than 500,000 sheep are inseminated annually in Australia, 300,000 in France, 60,000 in Spain and 50,000 in Canada (Hernández Ballesteros et al., 2015). In South America, AI in sheep has developed successfully in Brazil, Argentina and Uruguay and, in a much smaller scale, in other countries (Sepúlveda Becker, 2012).

There are several types of AI techniques, depending on the choice for semen deposition. Using fresh semen, by vaginal (pericervical deposition of semen), cervical (intracervical deposition of semen) or intrauterine (laparoscopic technique) deposition, satisfactory pregnancy rates are achieved (50-70%) (Faigl et al., 2012). However, by using frozen semen, laparoscopic or transcervical intrauterine insemination techniques are the only means to achieve acceptable pregnancy rates (Taqueda et al., 2011; Cseh et al., 2012; Kumar and Naqvi, 2014). As a reference, Taqueda et al. (2011) have obtained 45.8, 25.7 and 15.4% of pregnancy rates when applying intrauterine, deep cervical or cervical AI, respectively with frozen semen. Kumar and Naqvi (2014), using frozen semen by the transcervical technique, obtained pregnancy rates of 26 and 20% with two and one inseminations, respectively.

According to Fonseca et al. (2010), the expansion of the use of AI in sheep production requires generation of appropriate knowledge, adapted to breeds involved, as well as to the production system of small ruminants. There are some indispensable factors for a satisfactory result in AI, among them: female body condition, female prolificacy, nutritional management before and after insemination, synchronization and induction of estrus -rigor in the hormonal administration schedules-, male health, minimal stress in nutritional, sanitary and animal management. Male factors should also be taken in consideration such as age of the ram and mass motility of the semen used (David et al., 2015). Furthermore, it is important to take notes, especially referred to the seminal quality. Anel et al. (2005) also reported that farm, year and season, AI technique and technician are main factors affecting fertility after AI in dairy sheep. Recently, Errandonea et al. (2018) made an interesting reference about the importance of a short-term protein supplementation in the animal diet before cervical timed artificial insemination (TAI) with fresh semen, reporting an improvement in ovulation rate, prolificacy and fecundity. Satisfactory results after AI in dairy sheep are also influenced by meteorological variables, so that parameters such as temperature or rainy season can affect pregnancy success. The prediction of meteorological conditions could be a useful tool when AI dates are being scheduled (Palacios and Abecia, 2015).

The number of previous parturitions, interval from lambing to AI, reproductive seasonality, technician, farm and ram fertility are also factors affecting pregnancy rate (Palacín et al., 2012). In relation with the last factor, Vicente-Fiel et al. (2014) determined that high and low fertility rams present clear differences in several sperm quality parameters (sperm viability and motility, membrane integrity, sperm DNA fragmentation, sperm nuclear area, perimeter and length). Although this opens up the possibility of selecting males for AI based on sperm quality data, it is necessary to take into consideration that these parameters cannot be evaluated in field conditions. In contrast, Faigl et al. (2012) postulated that correlations between *in vitro* tests and fertility are not so high, considering qualitative parameters of the ejaculate (seminal concentration, wave motion, individual progressive forward movement) and more “elaborate” tests as termal resistance test, acrosomal integrity, ability of spermatozoa to travel in different media, hypo-osmotic swelling test, *in vitro* fertilization and computer assisted semen analysis (CASA). However, it was concluded later that sperm membrane integrity and straight-line velocity have a predictive capacity on field fertility (Santolaria et al., 2015). Unfortunately, fertilizing ability does not depend on a single parameter of the semen, but also on the fertility of the female, the type of estrus (natural or hormonally manipulated), the season and the site of deposition of the semen.

## Estrus induction and synchronization

Estrus induction and synchronization means carrying a group of females to the same stage of the estrous cycle, in general by means of hormone application; this procedure being a good tool for programmed reproduction especially for TAI. The TAI represents an important tool when detection of estrus is not feasible in commercial reproductive programs in small ruminants. An efficient TAI requires the use of hormonal protocols that ensure a high concentration of estrus in a short period of time in order to obtain acceptable pregnancy rates (proportion of pregnant females among all treated females).

Due to the application of a hormonal treatment, concentration of animals in estrus is achieved 24 to 72 h after hormonal treatment and ewes must be inseminated within a narrow lapse at the end of progestogen treatment. Moreover, AI takes full advantage of the estrus induction/synchronization technique as it allows accurate knowledge of the expected dates of parturition as well as the management of the lambing season (Faigl et al., 2012).

The most commonly used treatments for estrus synchronization are: intravaginal devices of progesterone (P_4_) release (CIDR) or intravaginal sponges impregnated with fluorogestone acetate (FGA) or medroxyprogesterone acetate (MAP), associated with equine Chorionic Gonadotrophin (eCG) near the end of progestogen treatment. In traditional treatments, progestogens are used for “long” periods similar to the lifespan of a cyclic corpus luteum, regardless of the stage of the cycle or the follicular status of the ovary at time of treatment initiation. dos Santos-Neto et al. (2015) showed a greater pregnancy rate with siliconed devices containing P4 than sponges containing MAP.

Menchaca and Rubianes (2004) in their review, summarized information and concepts in hormonal control of ovarian dynamics in small ruminants, supporting the use of progestogen and prostaglandin (PGF2α) for TAI in sheep. Also, they evaluated different alternative short-term progestogen treatments (so-called short treatments) in sheep, consisting of 5-7 days of progestogen priming. Menchaca et al. (2017) suggested that these short treatments result in a series of benefits like a better control of follicular response and ovulation, acceptable fertility rates (not lower than conventional P_4_ treatments), shorter period for implementation of large scale TAI programs, and in some cases, allowing the reutilization of intravaginal devices. In general, these advantages have important implications for the widespread adoption of insemination in sheep. In one of these trials on 1,750 multiparous ewes receiving intrauterine insemination, pregnancy rate was significantly higher with the short-term protocol than the traditional long protocol (43.5 vs. 37.8%, respectively; P<0.05). In another experiment in 922 ewes receiving cervical TAI with fresh semen, pregnancy success was also higher with the short-term protocol versus the traditional long protocol, using intravaginal devices of second use (41.2 vs. 29.1%, respectively; P<0.05).

The synchronization of estrus can also be achieved by the lysis of the corpus luteum, administering PGF2α. Recently, Fierro et al. (2013) published a review about the use of PGF2α and its synthetic analogues for controlling the estrous cycle of the ewe. In this review, the authors pointed out that the ovine CL is refractory to PGF2α treatment for up to two days after ovulation. They also pointed out that a second PGF2α injection improves estrus response when the stage of the estrous cycle at the first injection is unknown. Finally, they concluded that prostaglandin-based protocols for TAI might increase pregnancy rates by increasing the interval between PGF2α injections. Also, Fierro et al. (2016, 2017) published studies about ovarian follicular growth, concentrations of steroid hormones, estrous response, ovulation time, ovulation rate, conception, fertility and prolificacy rates after estrus synchronization with PGF2α, in order to identify which protocol was the most effective for TAI with fresh semen, establishing that the minimum length of the luteal phase prior to insemination, necessary for acceptable reproductive results, should be 12 days. Similarly, Fierro and Olivera–Muzante (2017) concluded that long intervals between PGF2α injections (15 or 16 days) determined better reproductive outcomes than mid intervals (12 or 13 days) after cervical TAI with fresh semen during the breeding season, equaling results achieved with the P_4_-eCG protocol. It is important to consider that in ewes synchronized with two PGF2α injections, 7 days apart, poorer reproductive performance was associated with an environment dominated by lower P_4_ concentrations, causing a preovulatory follicle with faster growth rate, larger maximum diameter and reduced oocyte competition. Furthermore, lower rates of ovulation, conception, prolificacy and fecundity were achieved (Fierro et al., 2011) when short PGF2α intervals were applied. However, de Carvalho Menezes de Almeida et al. (2018) reported that pregnancy and prolificacy rates were similar for PGF2α 7 days (73.3; 145%), 9 days (75.9; 125%), 11.5 days (75.9; 145%) in relation to the interval of days between PGF2α applications (P>0.05).

## Artificial insemination with fresh or cryopreserved semen (cooled or chilled semen)

In Argentina, AI in sheep is mainly performed with fresh semen. The insemination is carried out on synchronized estrus and the pregnancy rate is around 60% (Naim et al., 2009) -70% (Prieto et al., 2011), using sperm concentrations ranging between 60 and 100 total million sperm per ewe. The fresh semen must be used immediately after its collection, because its viability declines rapidly. The collected semen can be kept at 28-30 °C in thermostatic bath during insemination, either pure or diluted, for a period of time not exceeding 30 to 60 minutes, respectively.

The use of cryopreserved semen in sheep allowed to considerably accelerate the flow of superior genetic material towards population sectors of lower productive flocks, as well as to facilitate the transport of semen to farms near the AI center where rams are held. When cryopreserved semen is used, the ejaculate is diluted in order to provide an adequate means for its conservation. Dilution of semen is carried out for technical and biological reasons; technical reason has the purpose to increase the volume of the ejaculate to inseminate a large number of females (intensive use of the ram), while the biological reason has the purpose to provide the sperm with nutrients and protection against pH variations (Cueto et al., 1993). The most important recommendation is that all sheep should be inseminated with semen with wave motion (mass sperm motility).

The use of cooled or chilled semen (preserved semen, PS) in sheep is an economically viable practice, easy to implement, with acceptable and promising results. The AI with cooled (semen cryopreserved at 15 °C) or chilled semen (semen cryopreserved at 5 °C) is usually performed vaginally, by deposition of semen in the cervix, in the external uterine orifice. This route is the simplest and least expensive way to disseminate the use of semen in commercial flocks (Olivera et al., 2005). The PS in sheep allows seminal conservation and its subsequent use through vaginal AI, unlike what happens with frozen semen, which can only be used by the intrauterine route. By adapting estrus synchronization methods and AI protocols to the different production systems, it is possible to make an efficient use of the reproduction techniques, reaching acceptable pregnancy rates through the use of PS (40 to 55% with chilled and cooled semen, respectively) (Cueto and Gibbons, 2010). Future research will allow us to obtain protocols suitable for each region, promoting the dissemination of superior genetic material at a reduced cost for sheep smallholders.

The use of cooled semen is adequate for short periods of conservation (8-12 h) compared to the use of chilled semen, which is appropriate for longer periods (12-24 h) (Cueto and Gibbons, 2010). According to Dinatolo (2011), it is important to consider that preserved sperm present functional and structural changes in their membranes that determine a shorter half-life. In fact, the speed of sperm transport of chilled semen observed in the genital tract is lower than that of fresh semen (Fernández Abella et al., 2001). When semen was evaluated at 72 h after collection, there was evidence of better motility of ram semen after storage at 5 °C than after storage at 15 °C; however pregnancy rates with chilled semen decreased up to 72 h (60, 52, 30 and 18% for 0, 24, 48 and 72 h, respectively) (O’Hara et al., 2010). While others authors reported better quality sperm cells (significant differences for total motility, progressive motility, viability and acrosomal damage) after storage at 15 °C than after storage at 5 °C up to 48 h (Mata-Campuzano et al., 2014). Summary of some reproductive results in sheep obtained with AI with cooled or chilled semen are presented in Table 1.

**Table 1:** Summary of reproductive results obtained using preserved semen with different semen extenders and times of storage in sheep.

**Authors**	**Storage** **(ºC)**	**Preservation** **(h)**	**Semen Concentration (sperm million/dose)**	**Semen extenders**	**AI**	**Fertility (%)**	**Fertility statistics**
**Naim et al. (2009)**	5	12	150-300 x 10^6^	OviPro® (Biotay-Minitüb, Germany)	TAI^*^ (54-56 h)	32	a
		24				11	b
		12-24	300 x 10^6^			29	a
			150 x 10^6^			14	b
**Olivera et al. (2005)**	5	24	120 x 10^6^	TRIS (TRIS, fructose, citric acid, egg yolk)	AI (natural estrus)	19	a
		48				22	a
		24		“Piedra Mora” (UHT skim milk, egg yolk, glycerol)		49	b
		48				47	b
**Menchaca et al. (2005)**	5	12	200 x 10^6^	TRIS, glucose, citric acid, egg yolk	AI (natural estrus)	43	a
		24				35	b
		Fresh semen				54	a
**Cueto and Gibbons (2010)**	5	12	300 x 10^6^	skim milk, glucose	TAI* (52-56 h)	40	A review, no statistical analysis is presented
	15	6-8	150 x 10^6^	skim milk		55	
**Hozbor et al. (2009)**	18	8	---	UHT skim milk	TAI	47	

TAI*: timed artificial insemination in the external uterine orifice; hours (h) after sponge withdrawal;

TRIS: hydroxymethyl aminomethane; UHT: ultra high temperature; AI: Artificial insemination. Different letters differ considering P<0.05.

When analyzing available publications, there is great variability in the described results, possibly related to the different methodologies used: multiple diluents, natural or synchronized estrus, variable sperm doses and several preservation times.

## Frozen semen

Semen freezing techniques enable the conservation and national and international widespread of genes. When the semen is frozen and stored in liquid nitrogen (-196 °C), metabolic reactions of the sperm are stopped, allowing the preservation of seminal material for long periods. In this way, genetic material can be available at any time of the year. Furthermore, the use of germplasm of donor males, even after their death, is feasible (Aisen, 2004). The use of frozen semen has had a great impact on genetic improvement worldwide, by considerably increasing the flow of genetic material to general sheep flocks, as well as facilitating national and international transport and commercialization. By means of seminal freezing, expensive transport of males is avoided; while animal stress, physical and sanitary risk is diminished. This technique allows to preserve the genetic material of breeds at risk of extinction and to preserve genetic variability of breeds subjected to a continuous process of improvement of their productive characteristics.

Techniques for AI of sheep using fresh semen have been available since the 1960s, but the application of AI using frozen semen was hindered by the lack of methods for proper cryopreservation, storage and deposition of semen. Nowadays, the deposition of thawed semen directly into the uterine horns by laparoscopy observation results in good pregnancy rates that, in some cases, are almost equal to pregnancy rates obtained by vaginal insemination using fresh semen or by natural mating. This method has a success rate of approximately 60–75%, although this rate can vary depending on the quality of semen, the time of the year, the ewe’s body condition and the skill of the inseminator.

As mentioned previously, the use of frozen semen allows obtaining high fertility rates only by means of laparoscopic insemination; instead its efficiency through the cervical route is low and presents variable results (Faigl et al., 2012).

Salamon and Maxwell (2000) studied the possible causes of low fertility after cervical insemination with frozen semen in sheep and established two main limitations. The first limitation occurs as a consequence of the reproductive anatomy of the sheep cervix, whose cervical rings are more numerous and irregular than in other species; this condition reduces the effectiveness of cervical AI, as it constitutes a physical barrier to the deposition of semen into the uterus. The second limitation refers to the lower resistance of ovine semen to frozen-cryopreservation compared to that of other species. In this sense, it has been reported that ovine sperm are more sensitive to thermal stress by cold than those of other species such as rabbit or man (Garde López-Brea, 2002; Aisen, 2004; Dinatolo, 2011). Furthermore, even though addition of various antioxidants, hormones and other substances to freezing media have improved freezability of ram semen in experimental conditions, these results must be confirmed in field studies by lambing rates. The relaxin, oxytocin and PGF2α were used with two possible objectives, the relaxation of the cervix for the deep deposition of semen, or to increase the contractility of the genital tract to improve semen transport, but the use of these hormones did not present any beneficial effect (Salamon and Maxwell, 2000).

Several factors have been described as influencing pregnancy rates with frozen semen by laparoscopy. In general, pregnancy rates by laparoscopic insemination with frozen semen in sheep using AI after detection of estrus (65%) is higher than that obtained by TAI (50%; 60±2 h after removal of intravaginal sponges). This difference would indicate the importance of the moment of deposition of frozen semen in relation to the moment of occurrence of ovulation. Furthermore, this difference could be due to the reduced viability of frozen semen, which begins to decline from 6 to 12 h after insemination (Walker et al., 1989). The absence of differences in the fertility of natural estrus versus synchronized estrus suggests that the alteration of sperm transport due to hormonal treatment would not affect pregnancy rates, probably due to the intrauterine deposition of the seminal dose by the laparoscopic technique, close to the fertilization site.

Hill et al. (1998) presented results from AI with frozen and fresh semen considering the factors influencing pregnancy. The study comprised a high number of animals (28,447 Australian Merino ewes), using semen from 468 rams. The overall pregnancy rate was 71.7%. Pregnancy rates varied with type of progestogen implant, type and dosage of eCG, fresh or frozen semen, month of the year. The pregnancy rate obtained with MAP sponges (64.6%) was significantly lower than with 30 mg FGA (74.7%) or 40 mg FGA (72.1%) or CIDR implant (71.7%; P<0.05). An eCG dose of 200 IU resulted in significantly lower pregnancy rate (62.4%) compared with 250 IU (72.9%) or 300 IU (79.1%) (P<0.05). Ewes inseminated with fresh semen were significantly more likely to become pregnant (82.2%) than those inseminated with semen frozen in pellets (69.5%) or straws (71.6%) (P<0.001). Ewes inseminated during the months of March, April or May (fall, 71.5%) were just as likely to become pregnant as those ewes inseminated in November, December, January or February (69.6%) (P>0.05).

Since the 1970, efforts have been made in order to access the uterine lumen by the transcervical route (Fukui and Roberts, 1977). This technique allows the atraumatically deposition of the semen deeply into the uterine horns (Buckrell et al., 1994), resulting in a non-invasive practice. However, up to now, pregnancy rates obtained by transcervical insemination are generally lower than with laparoscopic insemination (fresh semen: 40-80%; frozen semen: 30-70%) (Cseh et al., 2012). In this sense, the process of manipulating an insemination catheter through the cervix has been linked to reductions in pregnancy and lambing rates (Kumar and Naqvi, 2014). On the other hand, insemination doses of 200×10^6^ (minimum) total spermatozoa are required. Cervical injury, abscesses, infections are also associated with the transcervical technique (Cseh et al., 2012). In addition, more time per inseminated ewe is required when compared to laparoscopy (~11 vs. ~57 ewes FTAI per hour for transcervical and laparoscopic insemination, respectively) (Casali et al., 2017).

In contrast, the laparoscopic technique allows the direct transfer of semen in the lumen of the uterus, achieving 60-80% pregnancy rates (Parkinson, 2009), with seminal doses of 40x10^6^ (minimum) total spermatozoa (Cseh et al., 2012). Disadvantages include the requirement of laparoscopic equipment, performance of invasive surgery and need for profesional labor (Cseh et al., 2012). Hill et al. (1998) postulated an increment in stress as the time interval the ewe remains restrained in the cradle increased. Therefore, animal welfare concerns must always be taken into account and both insemination procedures should avoid animal suffering as much as possible.

Boretto et al. (2002) published an important report concluding that the progressive individual motility test was the only seminal test that significantly contributed to the estimation of frozen semen fertility. This test has the advantage of being inexpensive, while providing an easy and quick methodology to evaluate semen quality, but it suffers the inconvenience of being a subjective evaluation. However, as long as the development of objective methodologies does not occur, mass sperm motility can be used as a sperm quality indicator, due to the positive correlation between subjective mass sperm motility and rate of fertility in field conditions (David et al., 2015).

## Conclusions

Long interval prostaglandin protocols followed by cervical timed artificial insemination is a cheap, green and easy-to-handle technique for genetic improvement in ovine productions, achieving pregnancy rates around 60 to 70%; however it is important to highlight that prostaglandin protocols can only be used in cyclic females.

The use of preserved semen for short periods of time acquires great interest in regional breeding programs especially considering the high demand of genetic material on a regional scale, low cost of the required equipment, practical procedure in semen management and feasibility for field implementation.

The widespread use of frozen semen in breeding programs in sheep still depends on more research for increasing reproduction efficiency, especially considering aspects related to cost/benefit and simplicity of the insemination technique.

In conclusion, regardless of the semen conservation and the technique used, artificial insemination is the method of choice for the rapid introduction/multiplication of valuable genes in an ovine population. All the recommended aspects should always be taken in consideration to ensure that the implementation of artificial insemination reaches the highest possible efficiency in order to obtain a significant number of offspring with high genetic merit and that justifies the technological investment in the production system.
